# Spatial and ontogenetic variation in isotopic niche among recovering fish communities revealed by Bayesian modeling

**DOI:** 10.1371/journal.pone.0215747

**Published:** 2019-04-18

**Authors:** Kyle J. Krumsick, Jonathan A. D. Fisher

**Affiliations:** Centre for Fisheries Ecosystems Research, Fisheries and Marine Institute of Memorial University of Newfoundland, St. John’s, Newfoundland and Labrador, Canada; Havforskningsinstituttet, NORWAY

## Abstract

Exploitation and changing ocean conditions have resulted in altered species interactions and varied population dynamics within marine fish communities off northeast Newfoundland and southern Labrador, Canada. To understand contemporary species interactions, we quantified the isotopic niches, niche overlap, and ontogenetic niche change among seven dominant fish species using stable isotope analyses. Analyses used fishes from three regions differing in fish and prey diversities. Differences in fish and diet composition diversity among regions were found using Simpson’s inverse diversity index. The regions of lowest diversities had higher instances of niche overlap and higher percentage of niche overlap area. The region of highest diversity had the widest spread of niches with greater distances from the community centroid. Ontogenetic shifts were observed such that larger individuals shifted towards the community centroid with the exception of Atlantic cod. Atlantic cod in particular was found to consistently be the top predator of the analyzed species. Our results reveal: (a) overlap in isotopic niches and spread within niche space was correlated with fish and diet diversity; (b) ontogenetic shifts are important when considering a species’ niche and quantifying spatial variation in community niche profiles.

## Introduction

Knowledge of diet and consumption by dominant predatory species is a key input to ecosystem approaches to fisheries management as species interactions are one of the main factors regulating fish populations [1–5]. In fisheries ecosystems of Newfoundland and Labrador, since the collapse of groundfish stocks in the early 1990s [5], numerous changes have been observed including rising ocean temperatures [6–7], a southward shift in Atlantic cod (*Gadus morhua*) and capelin (*Mallotus villosus*) distributions [8–10], and observed declines in Atlantic cod stocks followed by increases in snow crab (*Chionocetes opilio*) and northern shrimp (*Pandalus* sp.) populations [11–15]. Therefore quantifying spatio-temporal variation in feeding interactions and trophic structure studies is required to understand ecosystem functioning and predict future changes within this region.

The trophic niche is an essential component of a species’ ecological niche resulting from predatory and competitive interactions [16]. Trophic niches often vary throughout ontogeny as, for example, increased gape size allows for increased prey breadth [17–18]. In many cases body size, rather than species identity, predicts trophic position[18–20]. Stable isotope analyses have been proposed as a means to describe the trophic niche of species and communities by representing isotope data in multivariate space. Such portrayals are comparable to the n-dimensional space of an ecological niche [21–22]. However, the isotopic niche includes a combination of biotic and abiotic processes and thus is not the same as the trophic niche which result from the trophic interactions of an organism [22–23].The two measures are correlated as consumer-resource interactions are often a primary driver of isotopic niche [21, 24]

While stomach contents analyses have historically been used to describe diets, stable isotope analysis provides an alternative means of assessing energy flow through an ecosystem that integrates diet data over longer periods [25–26]. The stable nitrogen isotope signature (*δ*^15^*N*) typically becomes enriched by 3 ‰ for fish species with each consumption due to preferential removal of lighter amine groups during deamination, allowing for approximation of tophic level [27–29]. The stable carbon isotope signature (*δ*^13^*C*) provide an indication of the initial carbon source (pelagic or benthic in origin) and enrich at less than 1 ‰ with fractionation frequently considered negligible [30–32]. Four metrics relevant to quantification of community trophic structure using stable isotopes are presented below [23, 33]: Bayesian ellipses overlap, mean distance to centroid, mean distance to nearest neighbour, and standard deviation of distance to nearest neighbour. These metrics, in addition to providing details on trophic interactions, provide the foundation from which more complex food web dynamics models (e.g. [34–36]).

The coastal shelf ecosystems of Newfoundland and Labrador are considered recovering fish communities following overexploitation by fisheries and changing climate and ecosystem conditions [6, 15, 37]. The recovery dynamics, however, appear to vary among regions partially due to food limitations in the northern regions [10, 38–39]. This requires understanding feeding interactions within recovering fish communities and characterizing spatial differences among regions.

Towards this end, we use the four previously mentioned metrics of isotopic niche to analyze community trophic structure in recovering marine ecosystems. The specific objectives of this study are to: (a) determine the influence of species richness and diversity (both fish communities and their prey) on the community trophic structure among regions, and (b) assess the impact of ontogenetic variation on isotopic niche metrics.

## Materials & methods

### Study area

**Fig 1:**
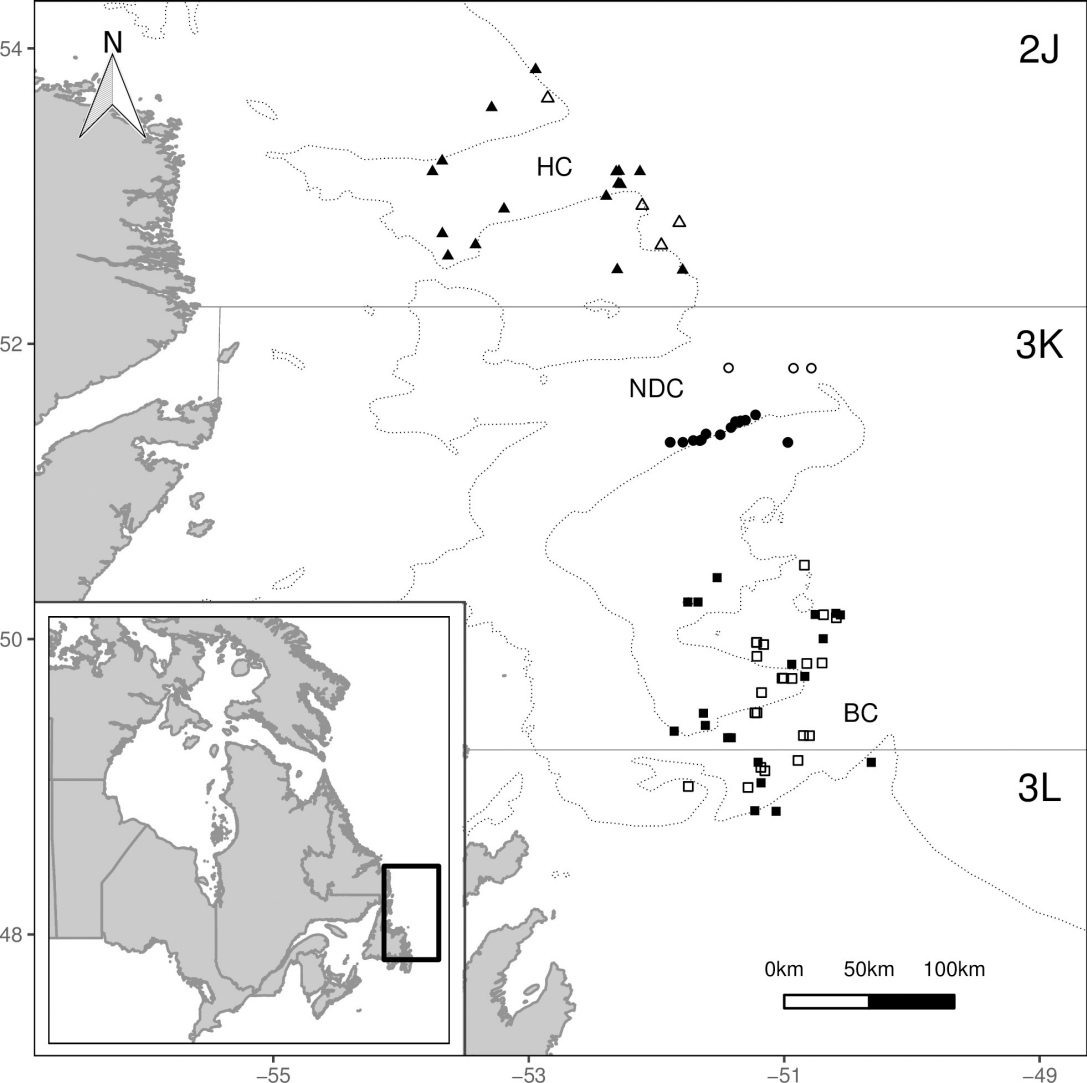
Map of newfoundland and southern labrador with sampling locations. The sampling locations are indicated for the 2013 (open symbols) and 2015 (closed symbols) locations within the Hawke Channel (HC, triangles), Notre Dame Channel (NDC, circles), and Bonavista Corridor (BC, squares). The inset map outlines the study domain in eastern Canada. The relevant NAFO subdivisions 2J, 3K, and 3L boundaries are also indicated. Dashed lines represent 300 m depth contours. The bathymetry map is reproduced from GEBCO world map 2014 (www.gebco.net) and NAFO subdivisions reproduced from NAFO (www.nafo.int).

The study was conducted as part of ecosystem surveys by the Center for Fisheries Ecosystems Research (CFER) aboard the RV Celtic Explorer in May 2013 and 2015 on the offshore shelves from southern Labrador and eastern Newfoundland, corresponding to Northwest Atlantic Fishery Organization (NAFO) subdivisions 2J and 3KL (Fig 1; for survey details, see [40]). These surveys were conducted in accordance with the Fishery (General) Regulations of Canada. As per section 52, experimental licenses were obtained from Fisheries and Oceans Canada (license numbers NL-1596-13 and NL-2927-15). The subdivisions 2J and 3KL together represent the management unit for the ‘northern cod’ stock of Atlantic cod (*Gadus morhua*). This region is dominated by the southward Labrador Current flowing along the shelf with sea surface temperatures steadily increasing with decreasing latitude [41–43]. Three major channels in these regions had previously been identified as important for onshore-offshore cod migration: the Hawke Channel, the Notre Dame Channel, and the Bonavista Corridor [44]. Given their potential importance, these trenches served as the focal regions for sampling cod and all other fish species within this study.

In both years a number of sets, defined as the catch obtained from a single trawl, were done in all three regions, though the Bonavista Corridor was more expensively surveyed. The trawl data for the fish species relative biomass composition were collected in May 2013 using a Campelen 1800 trawl (Table 1; Fig 1). While other trawl gear types were also used during this survey, the Campelen 1800 trawl sets from 2013 were chosen to represent the catch data as it was the only gear deployed in all three regions, allowing for regional comparisons. Samples for isotope analysis were collected during May, 2015, utilizing a combination of Campelen 1800 and mid-water trawls (Table 2; Fig 1). The variety of gear used for the opportunistic sampling of stomachs and samples for isotope analysis resulted from multiple projects being conducted aboard the survey. Given the short period between the two surveys in addition to low frequency trends in community composition across this region during this time period [15], the community composition is unlikely to have shifted substantially between the two years.

**Table 1:** Set details and diversity indices for 2013 Celtic Explorer data. Most abundant species (numbers, biomass) represents lists of the most frequently observed species pooled among sets within each region. Mean species richness and Simspon’s reciprocal were generated based on all trawl sets with regions.

Region	Number of Sets	Most Abundant Species (Numbers)	Most Abundant Species (Biomass)	Mean Species Richness (± SD)	Mean Simpson’s Reciprocal (± SD)
Hawke Channel	4	Redfish (26–88%)	Atlantic Cod (21–53%), Redfish (9–69%)	10.750 (± 2.754)	2.269 (± 0.992)
Notre Dame Channel	3	Atlantic Cod (4–65%), Redfish (24–83%)	Atlantic Cod (20–95%), Redfish (3–62%)	10.667 (± 2.081)	1.679 (± 0.352)
Bonavista Corridor	22	American Plaice (0–43%), Atlantic Cod (3–89%), Greenland Halibut (0–50%), Redfish (0–72%)	Atlantic Cod (1–99%), Greenland Halibut (0–60%), Redfish (0–96%)	13.212 (± 2.992)	3.588 (± 1.341)

**Table 2:** Set details and diversity indices for 2015 Celtic Explorer stomach data. Mean diet species richness and Simpson’s reciprocal evenness data based on pooled fish samples within regions.

Region	Number of Sets	Number of Stomachs Analyzed	Mean Diet Species Richness (± SD)	Mean Diet Simpson’s Reciprocal (± SD)
Hawke Channel	17	146	1.578 (± 0.846)	1.326 (± 0.515)
Notre Dame Channel	17	122	1.465 (± 0.731)	1.199 (± 0.384)
Bonavista Corridor	20	129	1.934 (± 1.149)	1.590 (± 0.835)

### Sample collection

All fish caught were sorted by species and their biomass was recorded. Sampled fish were swiftly killed with a sharp blow on the head. In 2015, fish selected for isotope analysis had their lengths measured for the following fish species, representing the most abundant species by sampled biomass: American plaice (*Hippoglossoides platessoides*, labelled ‘Plaice’ in figures), Atlantic cod (*Gadus morhua*, labelled ‘Cod’ in figures), capelin (*Mallotus villosus*), Greenland halibut (*Reinhardtius hippoglossoides*, labelled ‘Turbot’ in figures), lanternfish (N*otoscopelus* sp.), redfish (*Sebastes* sp.), and thorny skate (*Amblyraja radiata*, labelled ‘Skate’ in figures). We aimed to analyze twenty-one samples per species per region with as even a spread of sizes as possible. For species with little variation in size (capelin and lanternfish), only nine specimens were collected within each region. Based on the species’ observed length distributions, sampled individuals were classified as small, medium, or large, by dividing the observed range of sizes into three length categories of equal width (Table 3; S1 Table). These categories are recognized to be arbitrary, but as the exact timing of potential ontogenetic shifts was unknown, this division accounted for variation across the range of observed sizes. From most fish, a transverse sample of dorsal muscle tissue directly posterior to the head was collected, placed in 1.5 ml centrifuge vials and frozen at -20 ºC. Frozen stomach samples were also collected from these fish at sea. Remaining fish with small, difficult to sample stomachs were individually labelled bagged, frozen whole at sea and later dissected in the laboratory for their muscle tissue and stomachs.

**Table 3:** Definition of small, medium and large size categories for each species.

Species	Small size range (cm)	Medium size range (cm)	Large size range (cm)
American Plaice	7.0–22.6	22.7–38.3	38.4–54.0
Atlantic Cod	13.0–45.9	46.0–80.0	80.1–113.0
Capelin	11.0–13.6	13.7–16.2	16.3–18.8
Greenland Halibut	10.0–27.4	27.5–45.0	45.1–62.5
Lanternfish	12.9–14.5	14.6–15.6	15.7–17.4
Redfish	4.0–18.6	18.7–33.2	33.3–48.0
Thorny Skate	10.2–33.9	34.0–58.3	58.4–80.0

Size category definitions were consistent across regions. See S1 Fig for visual representation of species sizes.

Stomach content analyses identified and quantified the stomach contents to the lowest taxon feasible. Slow-dissolving features of prey, including otoliths, exoskeletons, and squid beaks were commonly used as prey identifiers. The mass and a count estimate of these prey were recorded with their identity.

Muscle tissue samples were oven dried at 75°C for 48 hours and homogenized using an amalgamator. The homogenized samples were weighed and analyzed at Cornell University Stable Isotope Laboratory (Ithaca, NY, USA). Approximately 1 mg of sample was placed into 7×7 mm tin capsules, then flash combusted using a Carlo-Erba NC2500 elemental analyzer coupled on-line to a Finnigan MAT Delta Plus mass spectrometer for analyses of the resulting carbon dioxide and nitrogen gases.

### Species richness and diversity

Two indices were used to describe the three analyzed regions. Both indices were applied to each survey set separately and averaged withinregion. Species richness is a count of the number of fish species present within a given set. The Inverse Simpson index [45], defined as follows:

$$\lambda =1/\sum_{i=1}^{S}{\left(n_{i}/N\right)}^{2}$$ (1)

Where *n*_*i*_ is the total number of a given species *i* in a set, *N* is the total number of all species in a set, and S is the total number of fish species within a given set. This λ represents a probability that two randomly chosen individuals will be of different species, such that higher values represent higher diversities.

The analysis of species richness and diversity were undertaken using data from the May, 2013, ecosystem survey aboard the same vessel and covering the same areas as the 2015 survey (Fig 1). These data were used to characterize richness and diversity (both fish in the community and diet) due to superior data on species at the level of individuals beyond the large demersal species specifically targeted in 2015. Only fish species were analyzed for species richness and diversity while invertebrates were also included for diet diversity measurements within each fish stomach. While trawl data may provide representation for larger species and individuals, smaller fish may be underrepresented. Stomach content analysis has been proposed for assessing abundance of species that may not be adequately represented in trawl data [46–47]. Therefore, to assess the differences in potential prey available across regions, the contents of a total of 397 (303 of which contained prey) stomachs from all seven species analyzed in the 2015 survey were analyzed. Species richness and An ANOVA was used to test differences between regions with response variables of species richness or Inverse Simpson’s Diversity and the categorical predictor variables of region and predator species.

### Stable isotope calculation

Nitrogen and carbon ratios were expressed in delta (*δ*) notation, being the parts per thousand deviation from the standard material: Pee Dee belemnite limestone for carbon and atmospheric nitrogen for nitrogen as follows:

$$\delta^{15}N\;or\;\delta^{13}C=\left(\left(\frac{\_R_{sample}\_}{R_{standard}}\right)-1\right)\times 1000$$ (2)

$$R={}^{13}C/{}^{12}C\;or\;{}^{15}N/{}^{14}N$$ (3)

Lipids were not removed to avoid the potential influence of derived products on isotopic signatures [48]. Therefore, following analysis, the *δ*^13^C values were normalized for lipid bias as recommended by [49], as follows:

$$\delta^{13}C_{normalized}=\delta^{13}C_{untreated}-3.32+0.99\times C:N$$ (4)

As the majority of fish samples were close to a C:N ratio of 3.3 as would be expected for muscle tissue of marine fish [49], this adjustment was only particularly relevant for lipid rich fish such as capelin, lanternfish and Greenland halibut.

Regional and ontogenetic variation was assessed using an ANCOVA on the following GLM:

$$\delta^{15}N\;or\;\delta^{13}C=Region*Length$$ (5)

With region being a categorical variable (n = 3) and length as a continuous variable. This analysis was conducted for each species separately.

### Bayesian ellipses

The remaining isotope analyses were conducted in two ways: (a) using size-pooled data as has frequently been done in such analyses and (b) splitting each species into three size categories to assess the influence of ontogenetic variation on metrics of niche overlap. The first of the Layman metrics was the Bayesian ellipse overlap, representing the core isotopic niche space occupied by a species. The construction of Bayesian ellipses, corrected for low sample size, was conducted in the R package SIBER [23]. Analyses were conducted among species, not among size groups within species. The standard Bayesian ellipses represent the core isotopic niche space represent bivariate standard deviation. The overlap between ellipses in isotopic space reflects overlap in the isotopic niches [23]. The proportion of overlapping ellipses is a count of the number of instances in which two Bayesian ellipses overlap in isotope biplot space over the total number of potential overlaps. The percent overlap is given by the percent of the overlapping area over the total area covered by the two ellipses. A mean of these overlapping areas was calculated for each region. An increased overlapping area represents increased isotopic niche overlap between the two species.

### Mean distance to centroid and nearest Neighbour

For the purposes of this study, the community centroid was considered as the mean carbon and nitrogen values of the centers of each species’ ellipse (sizes pooled). The distance to the centroid is therefore defined as the distance between the centers of each ellipse to the community centroid within each region [33]. This provides an indication of the degree of trophic diversity within a food web such that high mean distances indicate a wide variety of isotopic niches and low distances indicate a limited diversity of niches. The mean of these distances to the centroid was then calculated for each region. The distance to the nearest neighbour is calculated by analyzing the distance in biplot space between the center of a given ellipse and the center of each other ellipse of different species and selecting the shortest distance [50]. The mean of the distance represents the overall density of species packing in trophic niche space such that high values indicate wider spread within biplot space while lower values indicate higher density of niches and trophic redundancy. The standard deviation of the distance provide a measure of evenness of species packing in biplot space such that high values indicate skewed spreads of isotopic niches and low values indicate even spread of niches. The mean and standard deviation of the shortest distance to the neighbouring ellipses was then calculated for these measured shortest distances. A general linear model was constructed with response variables of either mean distance to the centroid or the mean nearest neighbour and the categorical predictor variable of region (n = 3). Bootstrapping was conducted on the mean and standard deviation distance to nearest neighbour to determine the credible interval around the calculated metrics.

## Results

### Species richness and diversity

No differences in the mean tow duration were observed among the three regions analyzed in the 2013 survey, allowing for comparison of the three regions (p = 0.28 from a one-way ANOVA). The Inverse Simpson’s Index based on fishes sampled, was found to vary significantly among regions (p = 0.05) with Bonavista Corridor being the most diverse and the Notre Dame Channel the least diverse (Table 1). The sets in the northern two regions were dominated by one or two fish species (Atlantic cod and redfish) based on percentage of total catch while sets in the Bonavista Corridor were characterized by a more even representation of fish abundances among species (Table 1). In contrast, mean fish species richness within survey sets did not differ significantly among the three regions (p = 0.25) (Table 1).

The Simpson’s Inverse diversity of the stomach contents was higher in the Bonavista Corridor than the other two regions (p < 0.01; Table 2), as was previously observed in these regions [51]. Similarly, prey species richness was significantly higher in the Bonavista Corridor than the two northern regions (p < 0.01). These trends were consistent across all species except the nearly-exclusively planktivorous lanternfish and capelin. A summary of these stomach content analyses are included in S2 and S3 Figs.

### Stable isotopes

**Fig 2:**
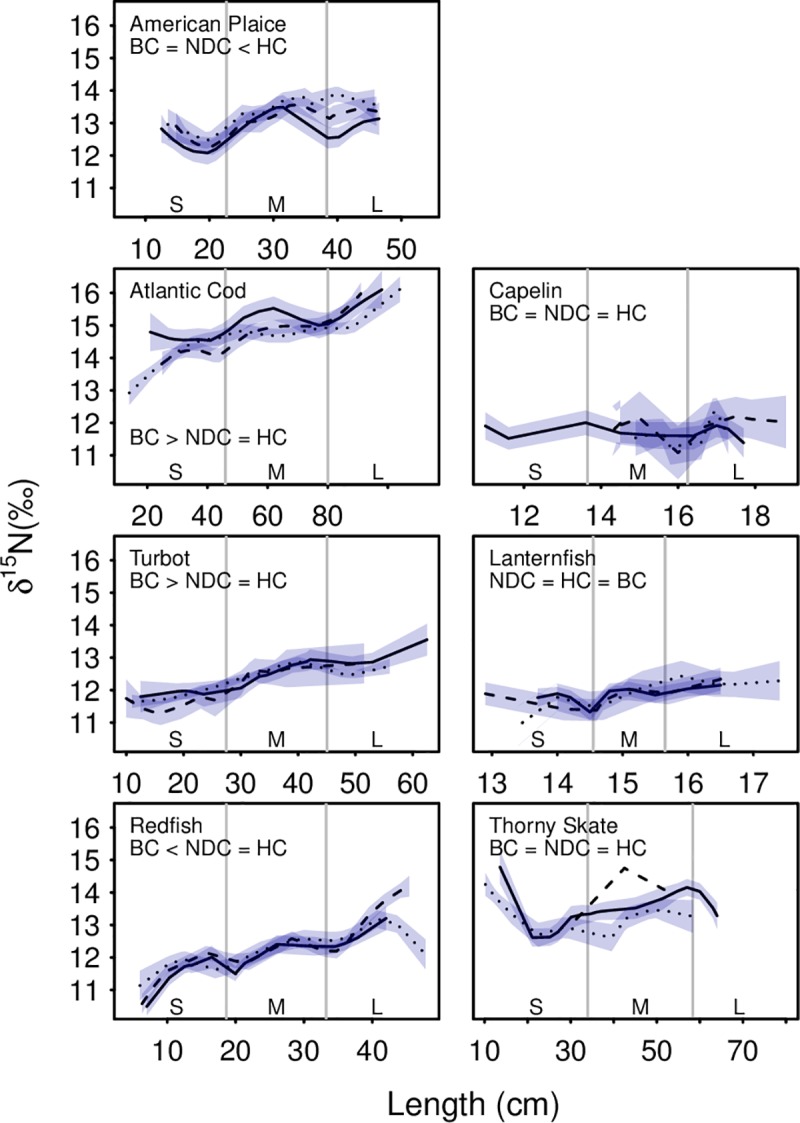
Nitrogen isotopic values across the seven species and three size classes (small, medium, large). Lines were fitted using local polynomial regression (α = 0.5) and the line type indicates region (solid: Bonavista Corridor; dashed: Notre Dame Channel; dotted: Hawke Channel). Error bars represent the standard error (except for Notre Dame thorny skate due to low sample size). For definitions of size categories, refer to Table 3.

**Fig 3:**
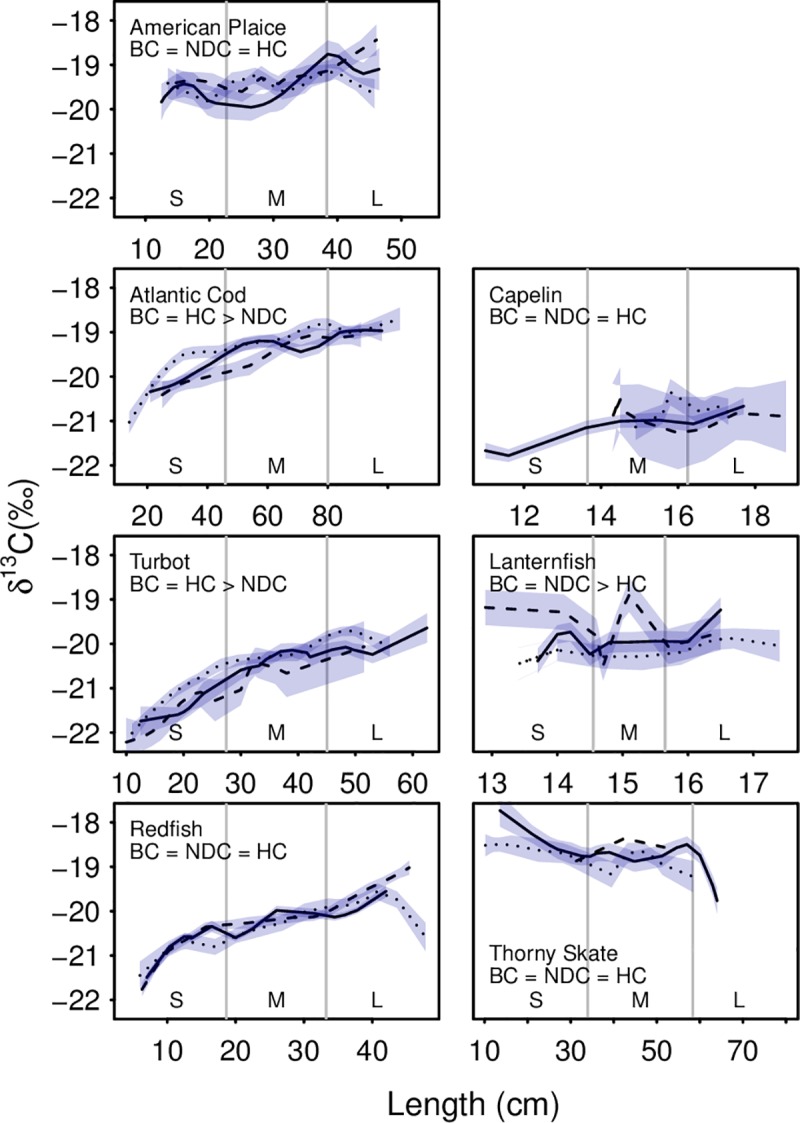
Carbon isotopic values across the seven species and three size classes (small, medium, large). Lines were fitted using local regression (α = 0.5) and the line type indicates region (solid: Bonavista Corridor; dashed: Notre Dame Channel; dotted: Hawke Channel). Error bars represent the standard error (except for Notre Dame thorny skate due to low sample size). For definitions of size categories, refer to Table 3.

Significant ontogenetic change in nitrogen signatures were observed in five of seven fishes examined (Figs 2 and 3). Nitrogen signatures increased with length for American plaice (p < 0.01), Atlantic cod (p < 0.01), Greenland halibut (p < 0.01), lanternfish (p < 0.01) and redfish (p < 0.01). As these species increased in size the nitrogen isotopic value increased indicating that larger individuals were feeding at a higher trophic level. Among regions, Atlantic cod (p < 0.01) and Greenland halibut (p < 0.01) were found to show significantly lower nitrogen signatures in the northern regions while American plaice (p = 0.01) and redfish (p < 0.01) exhibited higher values in the north (Fig 2). Capelin and thorny skate nitrogen signatures did not vary with length or region.

Carbon isotopic values generally increased with size for six of the seven species analyzed (Fig 3). With increasing size American plaice (p < 0.01), Atlantic cod (p < 0.01), Greenland halibut (p < 0.01), and redfish (p < 0.01) shifted from a zooplankton-dominated diet to a mixed pelagic/benthic based diet, while thorny skate (p < 0.01) shifted from a benthos-dominated diet towards a mixed pelagic/benthic diet. Atlantic cod (p = 0.02), Greenland halibut (p = 0.01) and lanternfish (p = 0.03) showed regional variation with their carbon signatures. Capelin carbon isotopic values did not vary with length or region.

### Bayesian ellipses

**Fig 4:**
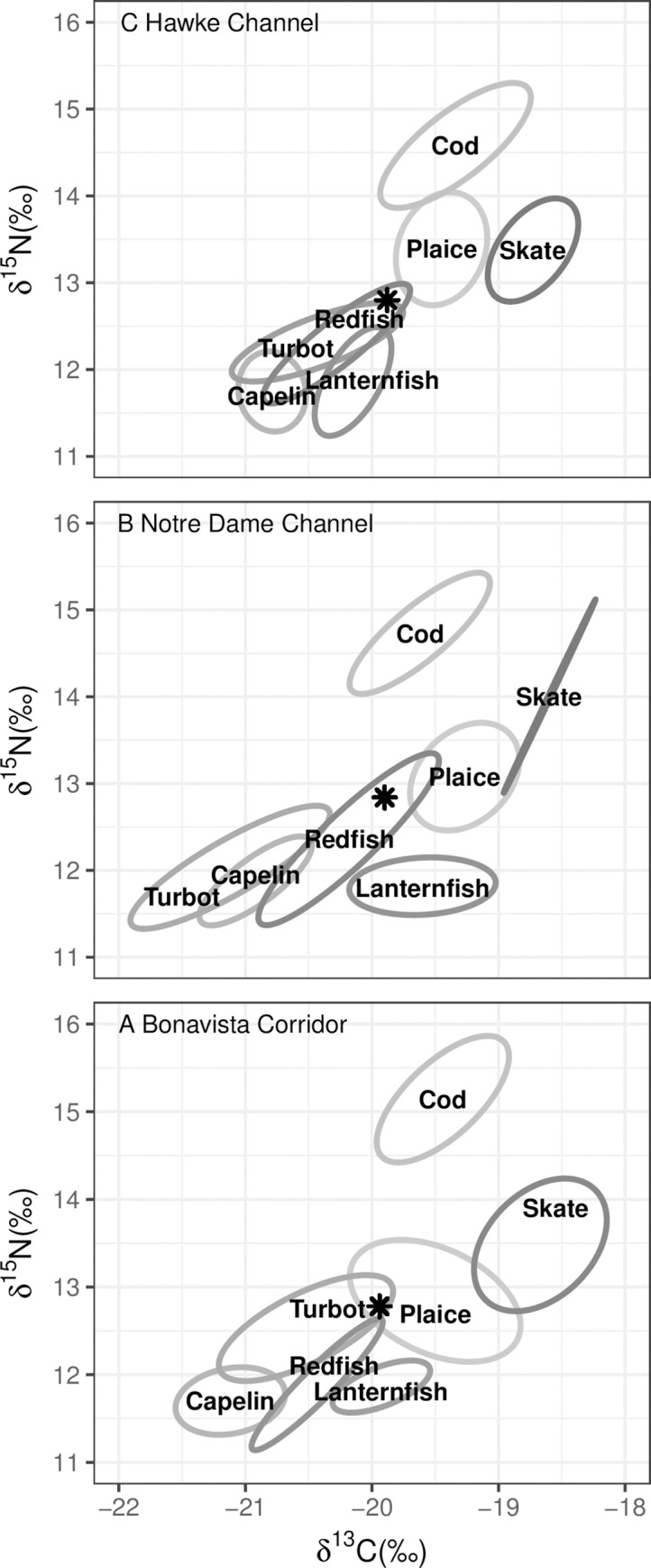
Bayesian ellipses for the seven species with all size classes combined. Individual panels represent: (a) Bonavista Corridor, (b) Notre Dame Channel, and (c) Hawke Channel. The star represents the community centroid within each region.

**Fig 5:**
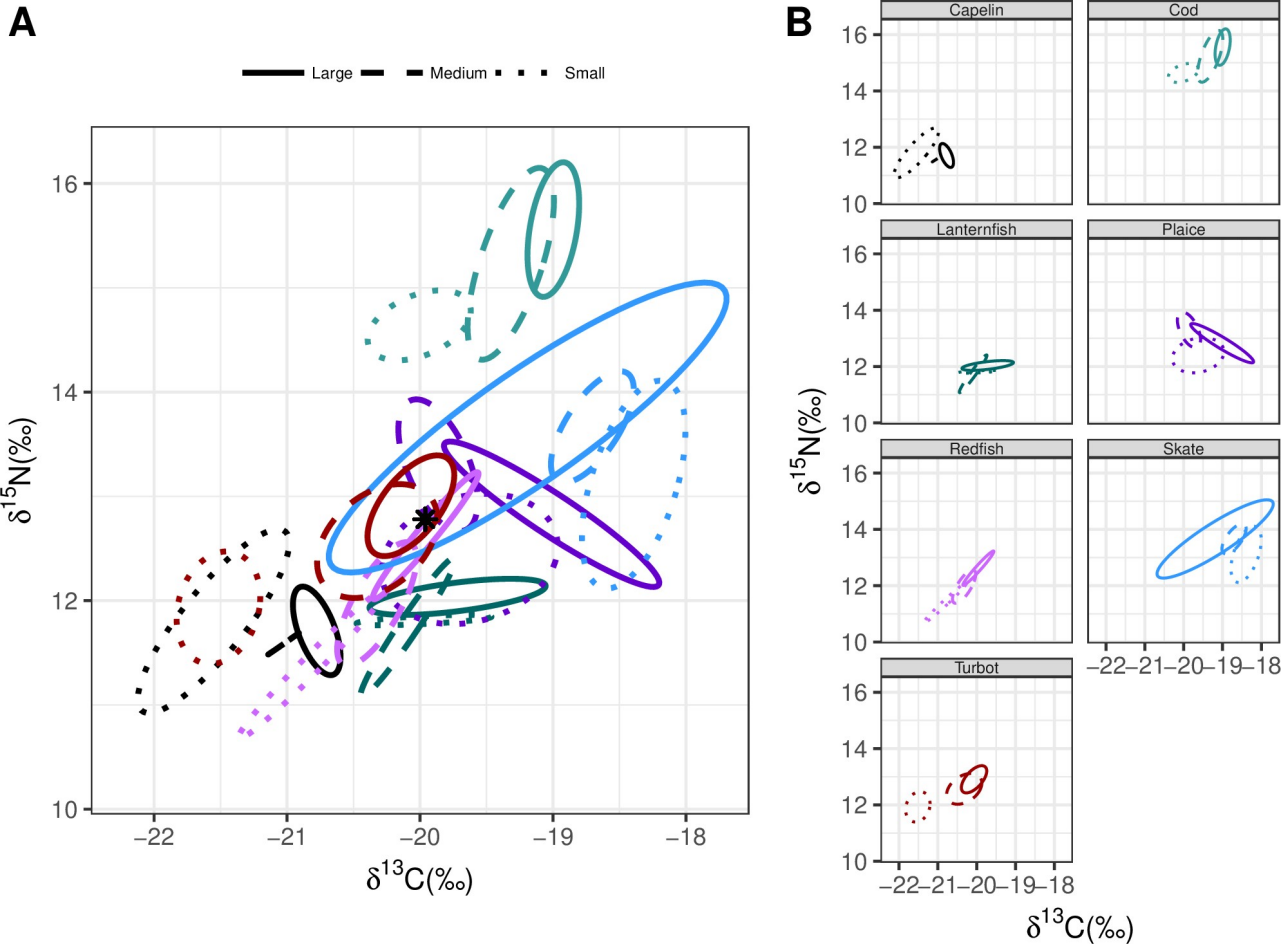
Bayesian ellipses for the seven species within the Bonavista Corridor separated by size class. The star represents the community centroid. Panel A shows the interconnection of species within the community and panel B shows the specific behaviours of each species with regards to size.

**Fig 6:**
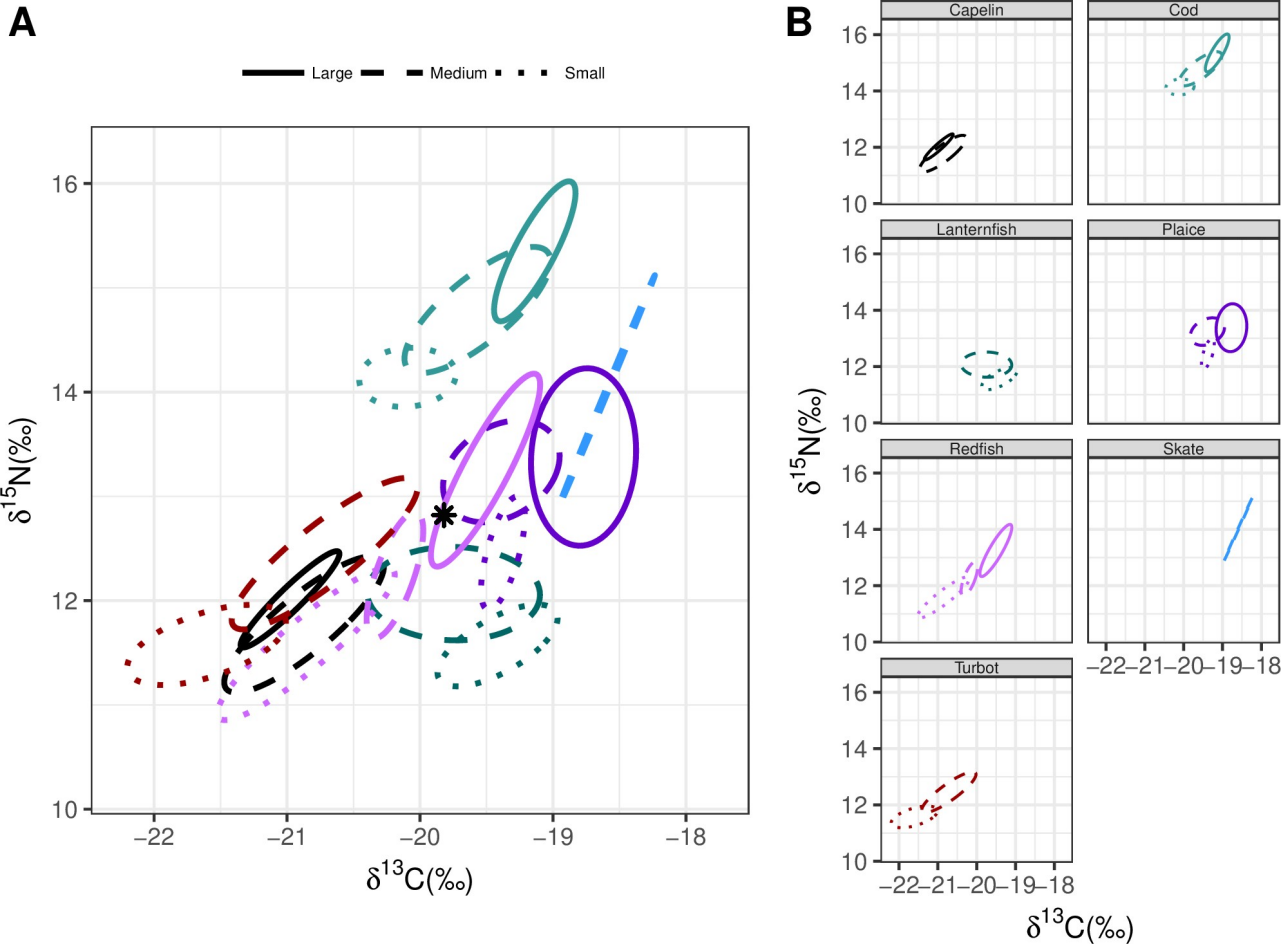
Bayesian ellipses for the seven species within the Notre Dame Channel separated by size class. The star represents the community centroid. Panel A shows the interconnection of species within the community and panel B shows the specific behaviours of each species with regards to size.

**Fig 7:**
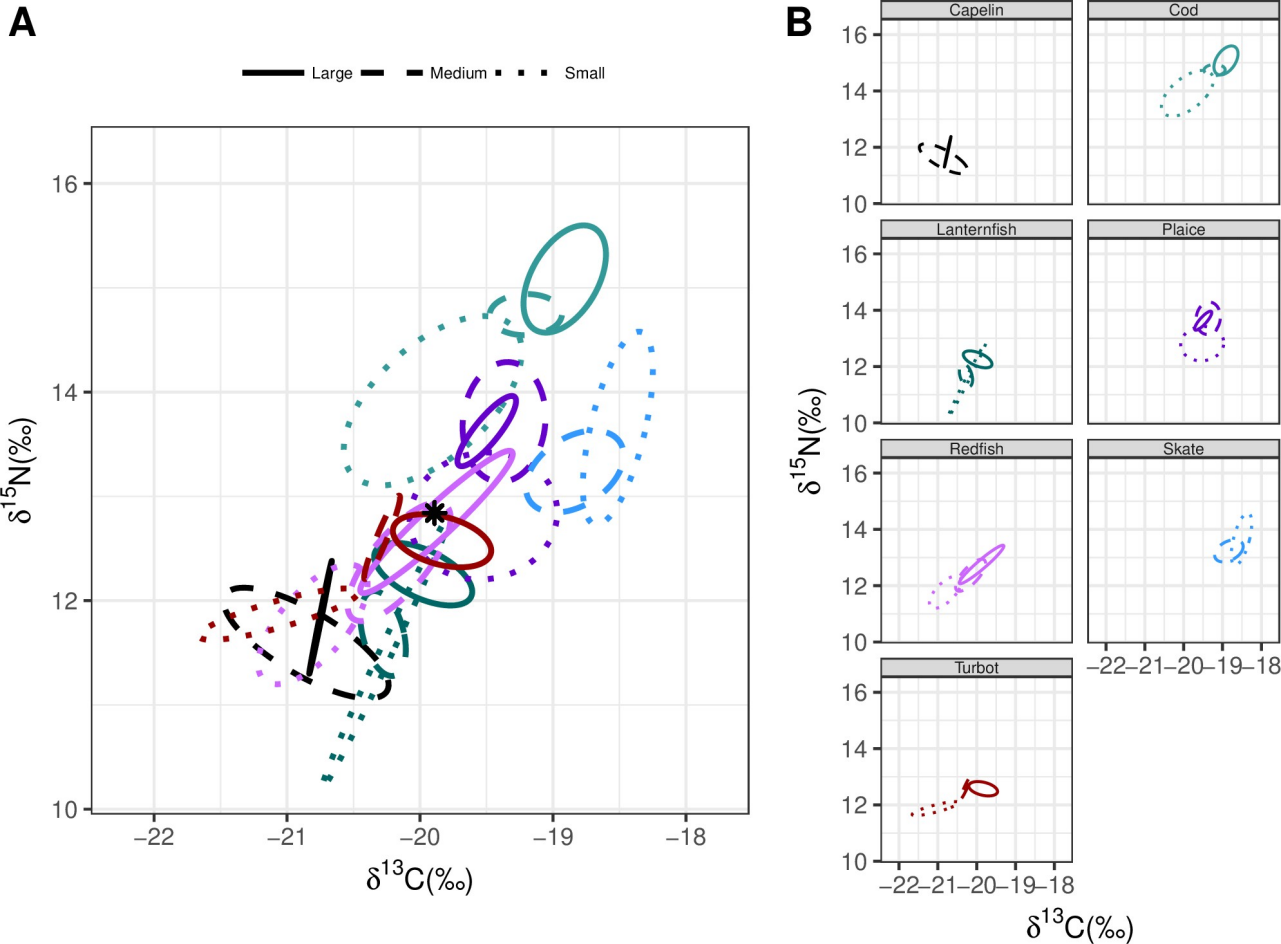
Bayesian ellipses for the seven species within the Hawke Channel separated by size class. The star represents the community centroid. Panel A shows the interconnection of species within the community and panel B shows the specific behaviours of each species with regards to size.

The proportions of overlapping Bayesian ellipses tended to increase with decreasing species diversity as did the percent overlap of these ellipses (Figs 4–7, Table 4). The Bonavista Corridor had the greatest spread of core isotopic niches in biplot space. However, in other regions, where prey diversity decreased, these core isotopic niches overlapped more and trended towards the center of the biplot, suggesting increased competition for less diverse prey resources (Table 4). Finally, the pooled and size-category separated analyses illustrated that while pooling all sizes within species together provided a general idea of the isotopic niche a species filled within the community, pooling increases perceived overlap in all cases (Table 4).

**Table 4:** The proportion of observed overlapped Bayesian ellipses, mean % overlap area of ellipses (± SE) and mean and standard deviation for distance to nearest neighbour (DNN; ± bootstrap CI).

Region	Sizes Separated				Sizes Pooled			
	Proportion of overlaps	Mean % overlap	Mean DNN	Standard Deviation DNN	Proportion of overlaps	Mean % overlap	Mean DNN	Standard Deviation DNN
Hawke Channel	0.23	2.07 (± 0.47)	0.46 (± 0.02)	0.37 (± 0.04)	0.36	4.01 (± 2.22)	0.58 (± 0.12)	0.37 (± 0.16)
Notre Dame Channel	0.19	1.88 (± 0.55)	0.58 (± 0.04)	0.39 (± 0.20)	0.24	1.47 (± 1.00)	0.81 (± 0.16)	0.42 (± 0.16)
Bonavista Corridor	0.16	1.52 (± 0.38)	0.58 (± 0.04)	0.47 (± 0.06)	0.27	0.84 (± 0.36)	0.90 (± 0.16)	0.47 (± 0.14)

Includes analysis for the exclusion (Fig 4) and inclusion of size categories (Figs 5–7).

### Mean distance to centroid

Region appeared to be important to explaining the distance of some species/size combination from the community centroid (Table 5), particularly Atlantic cod (p = 0.01) and capelin (p = 0.03). Species in the Hawke Channel were closer to the community centroid in almost all cases compared to those in the Bonavista Corridor. The Notre Dame Channel samples often exhibited erratic behavior that did not consistently follow trends of diversity, though overall the community tended to be closer to the centroid in regions with lower prey diversity. As species increased in size, capelin (p = 0.04), Greenland halibut (p < 0.01), lanternfish (p = 0.03), and redfish (p < 0.01) tended to gravitate towards the community centroid. Atlantic cod (p < 0.01) were found to stray away from the centroid with increased size.

**Table 5:** Mean distance to the centroid (± SE) by region for size-pooled (Fig 4) and size-divided ellipses (Figs 5–7).

Region	Small	Medium	Large	Pooled
HC	1.22 (± 0.40)	1.14 (± 0.43)	1.06 (± 0.46)	1.09 (± 0.17)
NDC	1.42 (± 0.53)	1.20 (± 0.45)	1.31 (± 0.49)	1.26 (± 0.19)
BC	1.46 (± 0.20)	1.22 (± 0.28)	1.07 (± 0.41)	1.21 (± 0.25)

HC standing for Hawke Channel, NDC for Notre Dame Channel, and BC for Bonavista Corridor.

### Mean distance and standard deviation to nearest Neighbour

The Hawke Channel fish species showed the closest proximity to their nearest neighbour and had the lowest standard deviation, indicating that individuals were more tightly packed within the isotope space, associated with increased trophic redundancy and/or competition (Table 4). The Bonavista Corridor showed a higher distance to the nearest neighbor and highest standard deviation, indicating the widest spread in niche space. In comparison to these regions, the Notre Dame Channel had the highest mean distance to nearest neighbour. The mean distances to the nearest neighbour generally decreased when considering within species sizes separated rather than pooled (similar to Bayesian overlap; Table 4). This similarity indicates that not incorporating size variation within species tends to inflate average distances among species (Table 4).

## Discussion

To understand interactions within recovering fish communities in Newfoundland and Labrador, we investigated how regional variation in fish and prey diversity, and fish size, influence four stable isotope metrics of community trophic structure. Trends in fish and prey diversity followed trends in the four metrics, indicating the potential importance of diversity in determining a species’ isotopic niche. Our study further quantified the importance of considering ontogenetic shifts in Bayesian ellipse analyses of community trophic structure.

Ontogenetic shifts in diet were found in nearly all species studied. Species that exhibited increased trophic levels with size, such as Atlantic cod and redfish, were found to incorporate more fish into otherwise invertebrate-dominated diets (S2 Fig). Pelagic feeders, such as redfish and Greenland halibut, incorporated more benthically derived prey into their diets with increasing size, frequently switching from diets dominated by zooplankton to incorporate more shrimp and small fish. Similarly, benthic feeders, such as thorny skate, showed more pelagically derived prey as demonstrated by increases/decreases in carbon signature, shifting from a benthic invertebrate dominated diet to incorporate more zooplankton. These observations are often consistent with trends observed in the stomach contents of these fish species (S2 Fig). Capelin, which did not vary with size in their carbon or nitrogen isotopes, demonstrated no change in their diet associated with size, consuming nearly entirely zooplankton at all observed sizes.

Numerous studies report ontogenetic shifts within a given species (e.g.[52–54]), yet few assess the importance of ontogenetic shifts on community structure (e.g. [55]). The vast majority of previous analyses that utilize stable isotopes and Bayesian ellipses group individuals together without intraspecific size considerations. If a species exhibits altered isotopic niches with ontogeny they would experience variable niche overlaps with different species [56]. While pooling the size categories by species may provide a general idea of the trophic role filled by the organism, such an approach will overlook complexities in ontogenetic niche shifts and competition across the life stages of the species. This pooling also leads to higher estimates of both proportions of niche overlap and mean percentage niche overlap vs. analyses that consider categories of fish size categories (Table 4).

An additional result of this study is that, with the exception of Atlantic cod, with increased size a species’ core isotopic niches trended towards a community centroid. With increasing body size gape size increases, expanding the range of feeding opportunities [17–18]. Increased size additionally allows for more efficient predation and generalized feeding, resulting in a trend towards to the centroid. These trends are also frequently associated with increases in the nitrogen signature, indicating an increased trophic level at which the predators feed with increased size. Atlantic cod, however, represents the top opportunistic fish predator sampled within this ecosystem and as such fills its own unique ecological niche [57–58]. It is important to note that both the 2013 and 2015 ecosystem surveys often targeted aggregations of dominant cod and/or redfish [40]. The diversity indices therefore may reflect the ecosystem as less diverse than what would expect from random sampling. This sampling pattern, however, was consistent across all sets. The variation observed in the diversity indices among regions therefore likely reflect underlying ecosystem variation.

Regional variation and related fish/prey diversity were related to isotopic niches in biplot space. The northern regions of this study (Hawke and Notre Dame Channels) exhibited reduced richness and evenness in both predator occurrences and predator diets. As such we would anticipate that species would be less capable of avoiding niche overlap as observed in the present study with an increased number and percent of overlap ellipse areas in northern regions. This would explain why the distance to the community centroid and mean/standard deviation of the distance to the nearest neighbor decreased between the Bonavista Corridor and the Hawke Channel and the degree of overlap of the Bayesian ellipses increased. The only species that were found to increase their trophic level in the northern regions was American Plaice, which was found to consume more demersal fish and crab in place of shrimp, and redfish, which was found to consume more shrimp and crab and less zooplankton. Species that demonstrated a decrease in trophic level, such as turbot, were found to consume fewer fish and more invertebrate prey (S3 Fig). Such local regional variation in *δ*^15^N is consistent with observations previously observed in this region [59], though reported for a wider variety of species in the present study.

Of particular interest to the Newfoundland and Labrador area are the variable observed recovery rates of Atlantic cod across regions. The Bonavista Corridor exhibited notable recovery rates, while the northern regions have experienced slower recovery rates as well as reduced growth and condition from their depletion during the late 1980’s and early 1990’s [40, 60–62]. The cod in the Notre Dame and Hawke Channels have been shown to frequently intermix and are genetically indistinguishable, yet the southern fish populations show minimal overlap with the northern populations and are genetically distinguishable [63–64]. Thus while the northern cod is managed as a stock complex [62], it contains a number of partially isolated subcomponents [65–66]. Recovery of these subpopulations could therefore come from two potential sources: recolonization from other subpopulations or resurgence of the local subpopulation [66–67]. In either case (or some combination of the two mechanisms), the subpopulations exhibit variability in the isotopic niche and presumably trophic niches.

Numerous other biological explanations have been provided for the variable recovery rates among these populations, including decreases in prey availability and increased predation [10, 39, 68]. Our results suggest another potential component: spatial variation in trophic overlap as evidence of higher potential competitive interactions in northern regions. In the Bonavista Corridor, Atlantic cod occupies a unique niche space at the upper part of the food web with no overlap in their core isotopic niche with any of the analyzed species (Figs 4–7). While still a top predator in the Notre Dame and Hawke Channels, Atlantic cod trophic level decreases. In the Hawke Channel in particular more ellipse overlap with other species is observed, particularly between juvenile cod and other species. In the Hawke and Notre Dame Channels, prior analyses from the years 1997 to 2011, consistent with the results of this study, have revealed that the diet of cod is heavily dominated by shrimp (55–99% of diet by weight) while fish species in the Bonavista Corridor make up a more substantial portion of the stomach contents (15–71% by weight) with overall higher fullness indices [51]. In all regions, as the cod increased in size they deviated away from the centroid and thereby reduced trophic overlap and potential competition with other species. The sizes that are most affected by this overlap would be primarily the small cod, which would include the juveniles and young adults. This feeding at a lower trophic level in small quantities relative to body mass [51] with increased isotopic niche overlap with other species together would represent an ontogenetic bottleneck which could limit the success of cod populations in the northern regions. Although competitive interactions are likely not the sole explanation for observed variability in recovery rates, it seems a potential factor.

Our results also illustrate how Atlantic cod fill a unique role within the fish community. While overlaps do occur with other species at smaller sizes in areas with reduced prey diversity, these fish appear to occupy a unique ecological niche. The other analyzed species typically exhibited much more substantial overlap in their Bayesian ellipses and presumably also in competition pressures. This unique position occupied by cod within the food web as a dominant fish predator could account for the cod’s success and abundance within this ecosystem prior to human exploitation.

There are survey spatial design issues that require consideration. For examples, there is little spatial overlap between the 2013 and 2015 survey in the Hawke and the Notre Dame Channels. However, given the consistent trends in diversity of the catch trends with the stomach data (Tables 1 and 2), the issue of spatial overlap is unlikely to have a significant impact on our results. In both years, the number of sets deployed in the Bonavista Corridor exceeded those in either the Hawke Channel or the Notre Dame Channel. Due to this limited number of sets, many of the isotope sample sizes were smaller, particularly in these northern regions. As the sampling was entirely opportunistic, sufficient sample sizes for certain size classes of some species (such as capelin) were simply not available. In cases were sample sizes were lower, two competing processes could influence our results, as demonstrated clearly with the case example of thorny skate. One could anticipate an increase in the size of the standard ellipses as the mathematical result of reducing sample size, as appears to be the case with large thorny skate in the Bonavista Corridor such that it occupies a large plot area (Fig 5). However, with decreased sample sizes could also underrepresent potential diet variability resulting in smaller ellipse areas, as is likely the case with thorny skate in the Notre Dame Channel where the ellipse appears almost as a straight line (Fig 6). Consideration of these errors presented here as examples serve to further support our hypotheses, as thorny skate overlap in the Bonavista Corridor would be overestimated and underestimated in the Notre Dame Channel.

Historically worldwide there has been little ecosystem focus in fisheries assessment and management decisions [69]. Single-species approaches have often failed and the state of many ocean ecosystems continued to decline [70–71]. Ecosystems, however, are complex adaptive systems such that understanding of the interconnection of components is essential in order to assess how species interactions and population dynamics will change [72]. Therefore investigating the interaction various components of an ecosystem is essential for ecosystem approaches within fisheries assessment and management [73–74]. Understanding multispecies trophic niche structure and how isotopic niches are associated with ontogenetic shifts may help reveal a species’ ecological role and what competitive pressures it may experience.

Knowledge of the present state of the ecosystem facilitates allows for the establishment of a baseline by which we can assess future potential environmental changes on isotopic niches. In the recovering and dynamic ecosystem we are presently observing in Newfoundland and Labrador, prey identities and quality are changing [75–77]. These studies predict an overall decreased prey field such that increased competition is likely to occur for a lower diversity of lower quality prey species. Should trends continue in this fashion, we would anticipate the regions occupied by Bayesian ellipses would trend towards increased interspecies ellipses overlap, as was observed in northern regions where recovery has not been observed [40, 62]. However, should ecosystem recovery occur approaching an unexploited ecosystem state with increased prey diversity, we might anticipate this trend to be reversed such that core isotopic niches within biplot space exhibit greater spread and decreased overlap with the isotopic niches of other species.

The regional differences characterized in this study follows trends observed in other marine ecosystems. The observed decrease in species packing with decreasing latitude as well as an increased overlap in Bayesian ellipses have been observed in other temperate to polar environments [78–79]. Furthermore, study regions at lower latitudes frequently showed lower degrees of trophic overlap (e.g. [54, 80–81]), though there are exceptions [82–83]. These studies did not connect any observed geographic variation to species richness or diversity but rather to factors such as latitude or salinity gradients (e.g. [78, 82]).

We have shown that niche overlap and potential competitive interactions, as revealed by each species respective positions in isotope biplot space, are associated with the biodiversity of available prey. With fewer options available, more overlap in biplot space was observed. With greater prey diversity species spread to fill the available isotopic/trophic niches. To further understand and establish a baseline for the present trophic structure of this marine ecosystem we require a more detailed understanding of predator-prey interactions that underlie these results, providing inspiration for future work presently underway.

## Supporting information

S1 Table Isotope sample sizes by region and size category with stomach sample sizes containing prey in parentheses. (DOCX)

S1 Fig Visual representation of size class definitions (small, medium, large) within and among species analyzed. (TIF)

S2 Fig Proportion represented by the Index of Relative Importance (IRI) of eight major prey groups by size category. Individual stomach contents were pooled by species and size category. The IRI is defined as IRI = (%N + %B)/FO, where %N is the percent by number, the %B the percent by biomass, and FO the frequency of occurrence [84]. Blue colours represent benthic prey while grey colours represent pelagic prey.
(TIF)

S3 Fig Proportion represented by the Index of Relative Importance (IRI) of eight major prey groups by region. **Individual stomach contents were pooled by species and region.** The IRI is defined as IRI = (%N + %B)/FO, where %N is the percent by number, the %B the percent by biomass, and FO the frequency of occurrence [84]. Blue colours represent benthic prey while grey colours represent pelagic prey.
(TIF)

S1 File Additional supporting data including set details, raw stomach data, overlap data of the Bayesian ellipses and the centroids of the ellipses. (XLSX)
