# Supplementary material for: Spatial and ontogenetic variation in isotopic niche among recovering fish communities revealed by Bayesian modeling
Source: PLoS One. 2019 Apr 18;14(4):e0215747. doi: 10.1371/journal.pone.0215747 (PMC6472828; doi:10.1371/journal.pone.0215747)
Supplement: S1 Table — (DOCX) [file pone.0215747.s001.docx]

|  | Region | Small | Medium | Large |
| --- | --- | --- | --- | --- |
| American Plaice | HC | 4 (4) | 10 (20) | 7 (2) |
|  | NDC | 5 (0) | 12 (2) | 4 (0) |
|  | BC | 7 (4) | 7 (7) | 7 (2) |
| Atlantic Cod | HC | 7 (3) | 8 (3) | 6 (2) |
|  | NDC | 7 (4) | 9 (8) | 4 (3) |
|  | BC | 7 (6) | 7 (7) | 7 (3) |
| Capelin | HC | 0 | 3 (2) | 5 (4) |
|  | NDC | 0 | 5 (14) | 4 (17) |
|  | BC | 3 (3) | 3 (6) | 3 (5) |
| Greenland Halibut | HC | 8 (10) | 7 (7) | 6 (2) |
|  | NDC | 8 (13) | 7 (2) | 1 |
|  | BC | 5 (3) | 9 (3) | 7 (9) |
| Lanternfish | HC | 3 (7) | 3 (15) | 4 (8) |
|  | NDC | 3 (3) | 4 (3) | 2 (2) |
|  | BC | 3 (5) | 3 (4) | 3 (3) |
| Redfish | HC | 5 (1) | 8 (13) | 8 (2) |
|  | NDC | 7 (9) | 7 (2) | 7 (3) |
|  | BC | 7 (3) | 7 (3) | 7 (1) |
| Thorny Skate | HC | 8 (5) | 9 (6) | 1 (1) |
|  | NDC | 0 | 3 (0) | 0 |
|  | BC | 7 (9) | 11 (14) | 3 (3) |
