# Supplementary figures and images for: Spatial and ontogenetic variation in isotopic niche among recovering fish communities revealed by Bayesian modeling
Source: PLoS One. 2019 Apr 18;14(4):e0215747. doi: 10.1371/journal.pone.0215747 (PMC6472828; doi:10.1371/journal.pone.0215747)

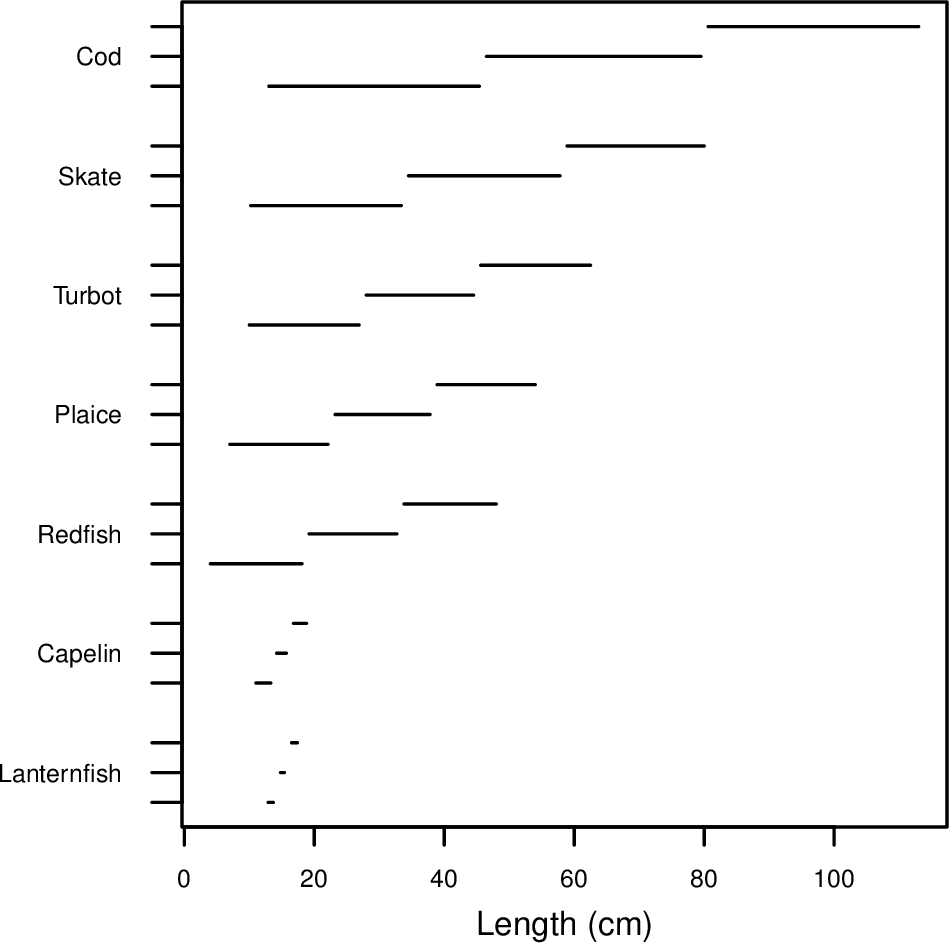

Supplement: S1 Fig — (TIF) [file pone.0215747.s002.tif]

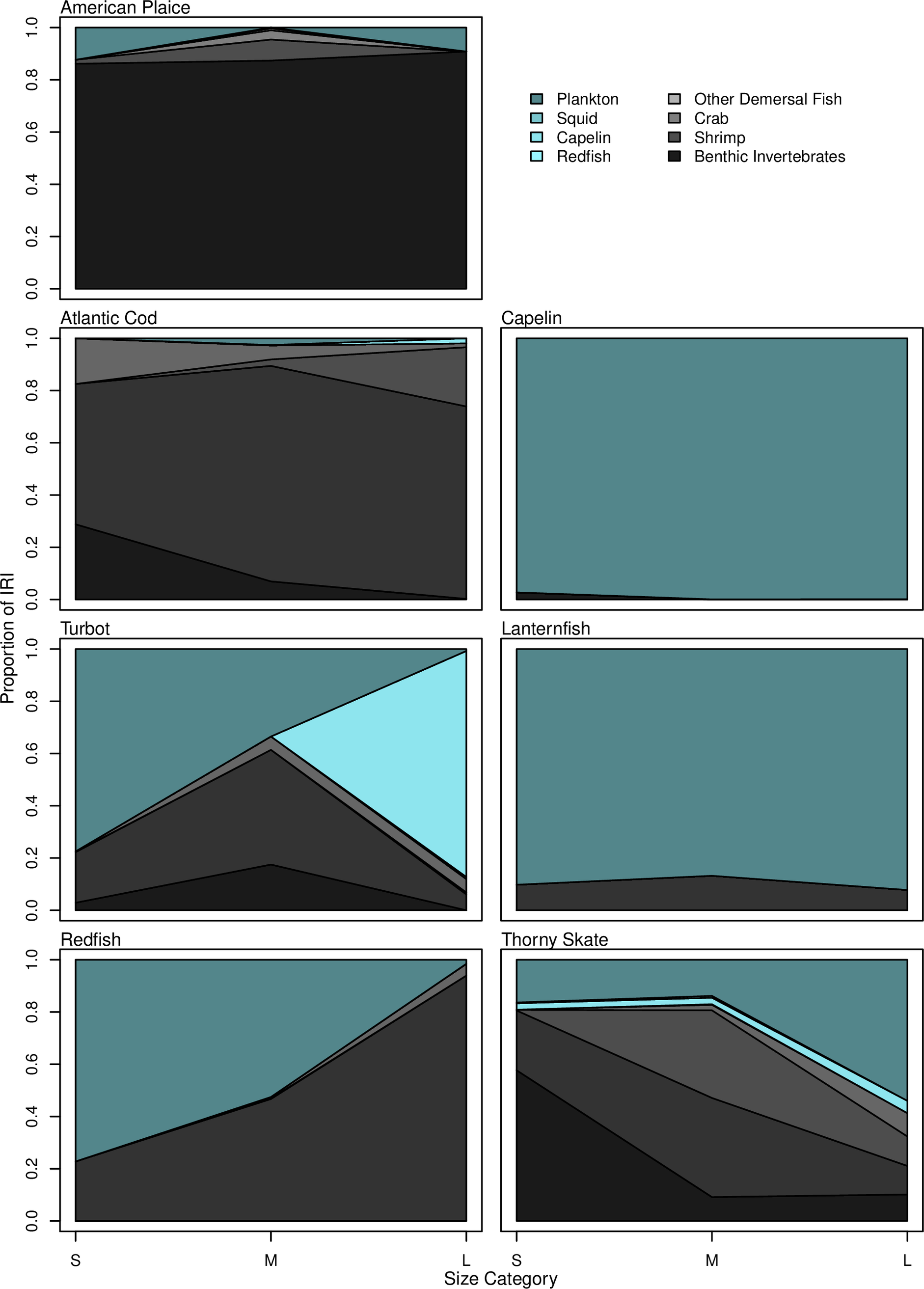

Supplement: S2 Fig — Individual stomach contents were pooled by species and size category. The IRI is defined as IRI = (%N + %B)/FO, where %N is the percent by number, the %B the percent by biomass, and FO the frequency of occurrence [84]. Blue colours represent benthic prey while grey colours represent pelagic prey. (TIF) [file pone.0215747.s003.tif]

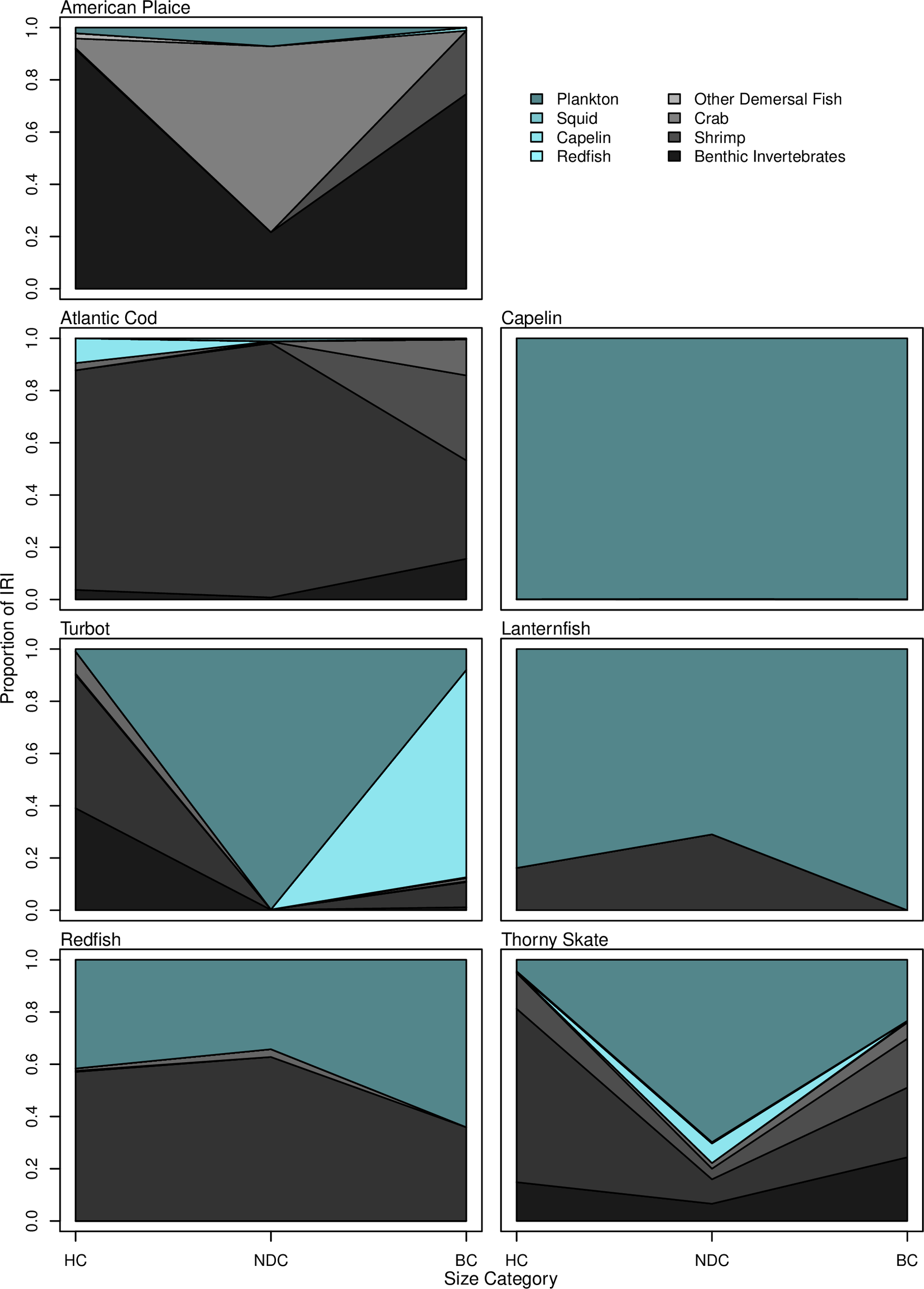

Supplement: S3 Fig — Individual stomach contents were pooled by species and region. The IRI is defined as IRI = (%N + %B)/FO, where %N is the percent by number, the %B the percent by biomass, and FO the frequency of occurrence [84]. Blue colours represent benthic prey while grey colours represent pelagic prey. (TIF) [file pone.0215747.s004.tif]
